# Coordinating culture change across the research landscape

**DOI:** 10.3389/frma.2023.1134082

**Published:** 2023-08-10

**Authors:** Leslie D. McIntosh, Cynthia Hudson Vitale

**Affiliations:** ^1^Digital Science, London, United Kingdom; ^2^Association of Research Libraries, Washington, DC, United States

**Keywords:** cross stakeholder coordination, misinformation, research integrity, disinformation, culture change

## Abstract

Scientific integrity necessitates applying scientific methods properly, collecting and analyzing data appropriately, protecting human subjects rightly, performing studies rigorously, and communicating findings transparently. But who is responsible for upholding research integrity, mitigating misinformation, and increasing trust in science beyond individual researchers? We posit that supporting the scientific reputation requires a coordinated approach across all stakeholders: funding agencies, publishers, scholarly societies, research institutions, and journalists and media, and policy-makers.

## 1. Introduction

Misinformation and disinformation proliferates worldwide with scientific research and communication offering no exception. Despite the best efforts of researchers, policy-makers, and others in the scientific ecosystem, scientific outcomes and recommendations can still mislead or be misled. As far back as 2013, the World Economic Forum cited the growth of misinformation and disinformation as a global risk especially in situations of high tension, when false information or inaccurately presented imagery can cause damage before it is possible to communicate accurate information (Howell, 2013).

The spread of misinformation is also directly related to public trust in research and science. A February 2022 Pew study on American confidence in groups and organizations found only 29% of U.S. adults say they have a **great** deal of confidence in medical scientists to act in the best interest of the public, while 78% have at least a **fair** amount of trust of that same group to act in the best interest of the public (Kennedy et al., 2022).

The reasons and motivations for lapses in research integrity and scientific malpractice are varied and complex. Integrity—as with most things—is a spectrum, with excellent research practice on one end and research misconduct (such as falsification, fabrication, and plagiarism) on the other. In the middle lies what may be a gray area of questionable research practices. When this spectrum is layered with stakeholders, such as journalists, funding agencies, and others, it becomes a matrix of motivations and outcomes. For example, a publisher may have the best intention to support scientific practices and increase research transparency, however, they may not have filters in place to prevent all nefarious acts, such as a confirmation bias that may surface through peer review or cursory quality checks on pre-prints. Moreover, a publisher or editor may feel a sense of duty to publish less mainstream science in order to push research forward (e.g., to some, acupuncture was fringe and could not be easily published for years). Perverse incentives that motivate questionable behavior in academia have been well-documented among researchers and academic institutions (National Academies of Sciences, Engineering, and Medicine, 2017).

The burden of improving scientific integrity most naturally falls on those individuals conducting research; yet their work does not occur in a vacuum. Additionally, individual stakeholder initiatives to improve science—while noble—can neither adequately support the research infrastructure, nor defend scientific integrity against coordinated attacks. Thus, the responsibility of upholding, fostering, and maintaining scientific integrity should rest on all stakeholders producing *and* consuming scientific information.

In their systematic review of research integrity literature, Bonn and Pinxten (2019) found the majority of empirical articles offering solutions focused on researchers' techniques and compliance—such as data sharing and open research practices. As the authors point out, the existing research system contributes and reinforces questionable research practices that undermine research integrity and negatively affects public perceptions of science and resulting policy. The authors also found that studies included in the literature review lacked addressing tactics or strategies to change the research system.

Valkenburg et al. (2021) unpacked a clear set of research integrity practices beyond individual and institutional responsibility. Specifically they addressed “culture and practices” with “four dispositions of doing, valuing, knowing and accounting” in the context of research integrity. While the authors do not explicitly mention stakeholders beyond individuals and institutions as bearers to uphold research integrity practices, they do allude to the broader research ecosystem and infrastructure as key to research integrity.

Worldwide, federal and private agencies are increasing their investments and recommitting to upholding the integrity of the scientific record in alignment with the Singapore Statement on Research Integrity [World Conference on Research Integrity (WCRI), 2010]. Labib et al. (2021) focusing on the relationship and incentives between research institutions and research funding agencies.

The Hong Kong Principles for assessing researchers (Moher et al., 2020) presents a model framework for assessing the integrity of research through the development of a set of indicators. These indicators cover five principles: responsible research practices; transparent reporting; open science (open research); valuing a diversity of types of research; and recognizing all contributions to research and scholarly activity. Efforts such as this are one step in addressing these system-wide challenges for research integrity—and are important for institutions, but leave out other key stakeholders in the research integrity endeavor—and are important for institutions, but leave out other key stakeholders in the research integrity endeavor.

Research integrity has been of critical importance worldwide. In their 2024 strategic plan, *Science, Technology, and Innovation Strategy for Africa 2024*, the African Union prioritized coordination among research stakeholders to promote ethics and research integrity. Similarly, the European Commission Horizon 2020 has invested over 20 billion euros in programs and initiatives to increase research integrity in programs and initiatives to increase research integrity since 2015 (Bonn and Pinxten, 2019). Within the United States, the recently released White House Office of Science and Technology Policy (OSTP) Science Integrity Task Force report (White House Office of Science Technology Policy, 2022) calls on policy-makers to “develop, implement, and [...] update scientific integrity policies.”

## 2. A coordinated approach to research integrity

Advancement in research integrity requires not only that we distribute and support responsibility *across* research stakeholders and disciplines to engender the culture change needed to proactively address issues of integrity, but also to embed research integrity at the core of research communications. It is not merely enough that a piece of research is completed with integrity, how research is communicated critically reflects that integrity and is itself an act of integrity. Because it is beyond the scope of this article, we are not looking at the granular nuanced differences across disciplines. Instead, we are discussing a view of collaboration and changes needed at the macro level in the scientific ecosystem. For us to progress, there needs to be a collective approach across the broader ecosystem of funding agencies, publishers, scholarly societies, institutions, journalism, and media, and policy-makers.

Important to this discussion is that of culture. Culture, in the context of this commentary, refers to the customs and practices of a community or social group. Specifically, we find that the current “culture” of science may be collaborative at the research level, yet is often disparate at the ecosystem level. It is this second aspect we are addressing, positing that disparate stakeholders and processes across the research integrity ecosystem need to increase coordination and communication.

The stakeholders involved in scientific communications processes have a unique incentive to engage in this coordination effort as each values and upholds the principles of research integrity. It would be naive not to acknowledge that each stakeholder may also have incentives to maintain the status quo and stay within the existing silos of scholarly communications. Nevertheless, the broader goal and vision will be significant enough to compel these stakeholders to work together to advance research integrity. Table 1 provides a summary of recommendations for each stakeholders.

**Table 1:** Summary of recommendations for stakeholders.

**Stakeholder**	**Role in ecosystem**	**Role in the perpetuation of good science**	**Recommendations**
Funder	Support well-conducted research and ensure funded research aligns with their organizational mission.	Clear expectations for reporting and sharing research.	**Examine** how their policies are implemented in practice; **Adjust** policies as necessary; **Support** the researchers with these processes when appropriate.
Publisher	Intermediaries in the dissemination of good science and offer space for full methodological processes to be described.	Publishing guidelines and checklists (e.g., STAR, Nature checklist).	**Move** information to more accessible formats; **Require** research transparency—moving beyond just data, into the sharing of full methods, interactive models, code, and software.
Scholarly societies	Provide a community of scholars aligned by a common discipline to offer support, opportunities for discussions, and guidance to members.	Setting expectations for reporting critical components of the research process, such as naming computer operating systems, code name and version, and even data availability.	**Coordinate** closer with other societies, publishers, and researchers, scholarly societies can support graduate students, faculty, and researchers.
Academic research institutions	Ensure prestige and ethics of research aligns with institutional mission and vision.	Providing support to ensure the institutions and their researchers comply with funding requirements, produce reproducible research, and uphold ethical standards of research.	**Assess** services and infrastructure available locally through University Libraries, campus IT, and Research offices; **Look** to external stakeholders for further collaboration.
Journalism and Media	Shape and influence public discourse.	Ensuring reporting is accurate.	**Conduct** research beyond the manuscript in question; **Verify** the research has been transparently reported.
Policy-makers	(Should) Make informed decisions based on science and balanced with public opinion.	Advocating and developing policies that uphold the integrity of science and research.	**Establish** more policies to increase trust and uphold the research integrity of science; **Follow up** those policies with assessments, modifications, and refinement to support the research enterprise.

**Funding agencies** support well-conducted research and ensure funded research aligns with their organization's mission. However, while stipulating many reporting and compliance requirements, these agencies generally do not say *how* to answer a problem or explore an idea. Because researchers and institutions—motivated to earn funding for research and to seek answers to challenging questions—will respond to agency policy requirements, funding agencies hold critical keys in fortifying the ecosystem.

Today, a growing number of funding agencies require proper data management and public access to articles. These requirements provide a necessary step toward transparency: We know that scientific advancement rarely comes from the results of one study, and research assets are continually reused and recombined to further science. However, most funding agencies only recommend and encourage data sharing—as opposed to requiring the sharing of research data, software, and other research outputs when feasible. To enhance trust in science and see the true impact of research investments, funding agencies should first examine how their policies are implemented, adjust policies as necessary, and support the researchers with these processes when appropriate. Moreover, they could consider tools to make science better and more manageable (such as protocols.io, Ripeta, or Vivli).

**Publishers** act as intermediaries in disseminating good science, offering space to describe robust methodological processes. Many societies and publishers work with the authors to improve the content quality and to better communicate the research. However, this stakeholder group also constricts communication through article formatting requirements, including limits on word counts, the number of citations, and sometimes the number of authors. In addition, formatting often restricts the full transparency of research components (e.g., research protocols, computational text, and interactive models).

Many publishers currently work to accommodate transparent and reproducible research and to evolve with changing needs and mandates. This support frequently comes in the form of publishing guidelines and checklists (e.g., STAR, Nature checklist). However, many publishers encourage depositing details or additional documents in supplemental files, which introduces discovery, reuse, and citation challenges. For example, moving detailed research methods or protocols to a supplemental file inhibits another researcher from applying the methods to a new data set and limits the citation of the original protocol. More than just presenting challenges for reuse, these practices limit science and the acceptance of these materials as first-class research objects. Publishers must further their push for research transparency and move beyond just data into the sharing of complete methods, interactive models, code, and software.

**Scholarly societies** provide a community of scholars aligned by a common discipline to offer support, opportunities for discussions, and guidance to members. Many manage one or more scholarly journals as part of broader missions to foster research, education, and scholarly cross-fertilization. They play an essential role in fostering trust in scholarship and connecting individuals to publishers and funders.

Unfortunately, support and guidance for reporting responsible research and data sharing are not widespread. Discipline and scholarly society communities can (and do) set expectations for reporting critical components of the research process, such as naming computer operating systems, code name and version, and even data availability. For example, the American Geophysical Union (AGU)^1^ has invested considerable time into understanding and communicating how to share and cite data, providing guidelines and support to members. Through coordination with other societies, publishers, and researchers, scholarly societies can support graduate students, faculty, and researchers in this critical research skill of the education and implementation of transparently reporting research. From an infrastructure perspective, this would be invaluable.

**Academic research institutions** care deeply about the integrity and quality of research produced by their scholars. They must ensure that prestige and research ethics align with the institutional mission and vision. As with other elements of the ecosystem, institutions need to understand and support the responsible reporting of research. Institutions are responsible for providing an environment and necessary support to ensure the institutions and their researchers comply with funding requirements, produce reproducible research, and uphold ethical research standards.

The Bonn PRINTEGER Consensus Statement: Working with Research Integrity- Guidance for research-performing organizations puts forth 13 key issues for research organizations to address to increase and uphold the integrity of research at their institutions. They include training and education, transparency of expectations, and aligning incentives (Forsberg et al., 2018).

Institutions should consider ways to support responsible research collaboratively and uphold research integrity while offering solutions to streamline and alleviate any unnecessary demands on researchers. Australian academic institutions have taken a model country-level holistic approach to support ethical and transparent research through supportive educational opportunities and interventions for researchers [Tertiary Education Quality and Standards Agency (TEQSA), 2022]. The shift here is not weighted toward supporting researchers rather than solely protecting the universities. Likewise, academic institutions should assess services and infrastructure available locally through University Libraries, campus IT, and Research offices and then look more broadly to external stakeholders and other academic institutions to collaboratively create solutions to increase research integrity.

**Journalists and Media** have a public responsibility and ethical requirement to ensure reporting is accurate and balanced. While journalists may be seen as outside of the traditional scientific communications workflow, with the advent of the internet and open science, journalists are an integral part of the new public and scientific discourse necessary for a well-informed community.

Given the important role that journalism has in shaping and influencing public discourse (e.g., COVID pandemic public discourse), the integrity and factualness of news articles is critical to uphold a well-informed community and create checks on federal and local policies. The last few years have seen a rise of news sensationalization among journalists and newspapers (Pickard, 2019; Lewis, 2020). While news views and reads are key indicators of article reach and impact, the drive to meet those needs should not outweigh the importance of accurate and thorough reporting.

Journalists should take additional steps to ensure that they are reporting on accurate research, such as verifying that the research has been transparently reported and investigating beyond the manuscript in question. As with researchers, though, responsibility also should be shouldered by their environment. News organizations and media platforms (including and especially social media) must support the resources and initiatives that can enhance trust in science communication and make journalism a trusted source of scientific information.

**Policy-makers**, traditionally beholden to all constituents, have a responsibility to advocate and develop policies that uphold the integrity of science and research. Additionally, policy-makers should make informed decisions based on science and balanced with public opinion.

A note of caution: Policies upholding research transparency should not override or be put before protecting privacy or confidential information. Nor should they place inappropriate requirements on federal agencies or others to prove facts unnecessarily. For example, toward the end of the Trump administration the Environmental Protection Agency was developing a set of rules titled, *Strengthening Transparency in Regulatory Science* (Environmental Protection Agency, 2018). While seemingly grounded in good practices for enhancing research integrity, the proposed policy would have in fact placed near impossible requirements for data and research sharing before policy decisions were to be made. Balance is critical.

While policy-makers may be federal agencies or funding agencies, they may also be local and federal congressional staffers or government officials. Thus, there may be some overlap between these two stakeholder groups. Developing policies and recommendations only for Federal Agencies and researchers is not enough, however. Additional best or effective practices for each of the stakeholders we've highlighted in this commentary is also critical to improve research integrity. More policy-makers must establish policies to increase trust and uphold the research integrity of science, and then follow up those policies with assessments, modifications, and refinement to support the research enterprise.

**Partnerships, coordination, and collaboration** among stakeholders are critical for the scientific integrity ecosystem and have grown in a number of promising initiatives in the recent years:

The Research Data Alliance (Research Data Alliance, 2023), a non-profit organization established 10 years ago, fosters data sharing and interoperability recommendations across disciplines and geographical borders. Through the RDA organizational platform, funding agencies, institutions, and publishers can join to align policies (Research Data Alliance Policy Alignment, 2023). This type of dialogue is critical for changing the world of research for two reasons: (1) It fortifies an informal infrastructure, which provides the opportunity for both stakeholders to raise the standards for research reporting while lifting the burden from researchers; and (2) will allow for a much-needed governance structure.

The Federal Demonstration Partnership (FDP) is a forum and point of collaboration between individuals from 217 universities and non-profits to work collaboratively with 10 federal agency officials to improve the national research enterprise [Federal Demonstration Partnership (FDP), 2023]. This organization has committees, subcommittees, and working groups focused on programmatic topics such as contracts, data stewardship, and finance/auditing/costing policies. Additionally, the FDP has developed standard templates for data use agreements, conflict of interest training documents, and more.

The Shorenstein Center on Media, Politics, and Public Policy within the Harvard Kennedy School, exists to also coordinate and facilitate understanding of “how people access, create, and process information” relating to “news and societal issues” *and* to offer solutions for problems (Shorenstein Center on Media Politics Public Policy, 2023). At the Center, workshops are hosted to “bring researchers in conversation with policymakers, journalists, and community organizers.” Since 2010, the Center has also worked to bridge understanding academic research for journalists through The Journalists Resource (Merrefield et al., 2023).

While the burden of systemic problems should not be shouldered by or faulted to individuals, organizations and funders can and should support individuals trying to effect change. Specifically, organizations must be aware of how organizational changes via individual initiatives may dissolve without their chief advocate.

To truly move forward and effect change, integral changes strengthening integrity need to be embedded within and across these organizations. Systematic change comes when all players in the ecosystem work toward a common goal. Organizational stakeholders must coordinate efforts to fortify science integrity: making science better, and better science easier.

## Data availability statement

The original contributions presented in the study are included in the article/supplementary material, further inquiries can be directed to the corresponding author.

## Author contributions

LM and CHV both contributed equally to the design, drafting, and finalization of the article. They shared contributions. All authors contributed to the article and approved the submitted version.

## Footnotes

^1^Data and software for authors. Available online at: https://www.agu.org/Publish-with-AGU/Publish/Author-Resources/Data-and-Software-for-Authors (accessed March 27, 2023).
